# Satellite and UAV Platforms, Remote Sensing for Geographic Information Systems

**DOI:** 10.3390/s22124564

**Published:** 2022-06-17

**Authors:** Alfred Colpaert

**Affiliations:** Department of Geographical and Historical Studies, University of Eastern Finland, Yliopistokatu 7, P.O. Box 111, 80101 Joensuu, Finland; alfred.colpaert@uef.fi

Satellite and UAV (unmanned aerial vehicle) imagery has become an important source of data for Geographic Information Systems (GISs). Enormous volumes of remote sensing and GIS data are nowadays produced, stored and analyzed in high-capacity network-based geoinformatics systems. Satellite imagery in a wide range of spatial, spectral, and temporal resolutions provides the scientific community with rapidly available global data to be used as an integral part of spatial data structures and analyses. Remote sensing platforms, such as Modis and Landsat, have a unique historical record of providing tens of years of uninterrupted global data, provided by repositories such as NASA and ESA.

For local applications, the rapid evolution of unmanned aerial vehicles and lightweight sensors has provided the scientific community with a tool for acquiring data of extremely high resolution, covering areas that vary from several hectares to hundreds of square kilometers in size. These data can be used for precision farming, forestry, and environmental monitoring.

The present Special Issue on the integration of UAV and satellite imagery with GIS contains ten articles, which can be divided into three parts: UAV applications, satellite remote sensing and methodological work using RS data.

Three articles deal with pure UAV applications, two applying UAV for agricultural crop monitoring [1,2] and one paper [3] using GIS and computer vision to analyze UAV orthomosaics. The planning of high-altitude long-endurance pseudo-satellite missions is dealt with in this paper [4].

The integration of satellite RS data in GIS systems for vegetation monitoring is used in three papers: one paper dealing with winter stress on arctic understory vegetation [5], one on the application of Copernicus (CMEMS GlobColour-Merged CHL-OC5 Satellite Observations) satellite-derived data concerning the aquatic environment [6] and one on the application of MODIS NDVI data and GIS to assess the effect of wildlife upon tropical savannah vegetation [7].

The problem of mosaicking multiple high-resolution orthoimages (UAV or satellite) is dealt with in paper [8], introducing a novel method based upon the D-LinkNet Neural Network. 

Precise satellite telemetry data can be used to monitor the minute deformations occurring in the Earth’s crust. The paper by Jagoda et al. describes the use of high-precision laser altimetry data to assess the deformation of the Earth’s crust caused by external planetary bodies (tidal forces) [9]. The last paper uses data derived from global navigation satellite systems (GNSS) to monitor the crustal deformation of the Earth [10].
